# Editorial: Understanding the impact on metabolic health of interactions between pre- and post-natal nutrition, sex, growth and endocrine development

**DOI:** 10.3389/fendo.2024.1503983

**Published:** 2024-10-29

**Authors:** Anne L. Jaquiery, Mark H. Vickers

**Affiliations:** Liggins Institute, The University of Auckland, Auckland, New Zealand

**Keywords:** prenatal nutrition, postnatal nutrition, interactions, sexual dimorphism, metabolism, DOHAD

There is now widespread acceptance that the preconception, fetal and early postnatal environment shapes the development of physiological systems that determine growth and metabolism. Barker and Thornburg (2013) expanded on the ‘developmental origins of health and disease’ (DOHaD) paradigm, suggesting that the pathogenesis of chronic disease is better understood as the product of branching paths of development after environmental insult rather than an additive response to adverse influences at different stages of life (1). The nutritional environment is clearly key; however, it is likely that metabolic health outcomes arise from complex interactions between nutrition, genetics, epigenetics and physiological systems, including neuroendocrine pathways and the more recognized influences of stress axes and the gut microbiome.

Basic science research continues to contribute to understanding of the mechanisms underlying the influence of the nutritional environment on development during fetal and early postnatal life. However, experimental work often focuses specifically on one aspect, such as the effect of nutrition on pancreatic development and subsequent glucose/insulin metabolism, or the genetics of growth-related hormones, rather than the interactions between them. One important interaction which has been increasingly explored over recent years is the relationship between early life nutrition, metabolic outcomes and fetal sex.

Many developmental programming studies have found that male and female offspring exhibit different phenotypes following an adverse *in utero* environment (2). In the past, experimental studies of developmental ‘programming stimuli’ in both small and large animals were typically performed only on either males or females, or did not have adequate power to analyze the sexes separately. It is therefore likely that some key physiological interactions were missed - for example, if opposite effects occurred in males and females in response to the same intervention, analysis with the sexes combined would likely have ‘cancelled out’ and masked potentially important differences. This was demonstrated in a study of postnatal nutritional supplements in lambs, in which insulin response to intravenous glucose tolerance test was increased in males but decreased in females, compared to same sex controls. Similarly, mRNA expression of key genes in β-cell development showed sexually dimorphic effects (3). Work in the mouse has also shown that maternal intake of the artificial sweetener acesulfame-K results in sex-specific alterations in adipogenesis and glucose tolerance in offspring (4). Sexual dimorphism may also explain seemingly contradictory results across experimental studies which focused only on one sex.

The basis for the observed sexual dimorphism in ‘programming’ of the metabolic syndrome is still not well understood (2). It remains unclear what aspects of the differences between male and female development give rise to the differential susceptibility to programming insults. Similarly, an increasing number of studies have demonstrated that males and females respond differently to nutritional interventions designed to improve long term metabolic outcomes after *in utero* or postnatal compromise. More recently, researchers have sought to fill this knowledge gap. It is therefore not surprising that several of the articles submitted for this Research Topic explored the impact of fetal sex on nutrition, development and later health outcomes.

Estrella et al. investigated whether sex differences in prenatal growth were likely contributors to sex-dependent programming effects on postnatal phenotype. They approached this by integrating data from several scientific modalities in the mid-gestation bovine conceptus. They were able to demonstrate that the female conceptus displays asymmetric lower growth compared to males, implicating female-specific modulation of key endocrine mediators by nutrient supply. They hypothesized that this mode of female development may increase resilience to environmental perturbations *in utero* and contribute to sex-bias in programming outcomes, including susceptibility to non-communicable diseases.

Christians and Reue reviewed the available literature exploring the role of gonadal hormones and sex chromosomes in sex-dependent effects of early nutrition on metabolic health, emphasizing the important distinction between chromosomal and gonadal sex. The authors make the point that elucidating the relative roles of chromosomes and hormones on metabolic health ‘programming’, and identifying patterns that emerge – for example, the relative responsiveness of one or the other to prenatal insults - might open potential avenues for nutritional or other interventions, which may need to be sex-specific.

Fetal sex has also been shown to influence the maternal metabolic milieu during pregnancy. This has mainly been explored in relation to glucose metabolism and gestational diabetes. In their article for this Research Topic, Wu et al. considered whether fetal sex was associated with maternal thyroid function during pregnancy. The authors found a sexually dimorphic profile of maternal thyroid hormones late in the first trimester, with TSH concentrations lower and thyroxine concentrations higher in women carrying a female fetus and hypothesized that this could be related to the observed higher HCG levels associated with a female conceptus.

These three articles add valuable new information to the increasing body of literature focusing on interactions between fetal sex and the nutritional and hormonal environment during pregnancy.

The final two articles in this Research Topic examined associations between nutrition, growth and metabolism. Zhao et al. investigated the relationship between cord blood vitamin A and vitamin D levels and postnatal growth to 6 months of age. The authors found a positive relationship between Vitamin A levels at birth and head circumference growth, and between cord blood vitamin D levels and BMI growth, in infants aged 3–6 months, suggesting an interaction between prenatal nutrition and postnatal growth.

Nyen et al. examined the association between *in utero* exposure to maternal glycaemia and offspring glucose metabolism at 9 years of age and found a positive association between maternal and offspring fasting glucose, which persisted after adjustment for sex and potential hereditary and lifestyle confounding factors.

This Research Topic aimed to combine knowledge from existing and new studies to increase understanding of interactions between pre- and post-natal nutrition, sex, and physiological systems that contribute to healthy or adverse metabolic outcomes. Such information is crucial for targeted development of effective interventions that may need to be sex-specific and will contribute to the development of integrated ‘packages of care’ in fetal and early postnatal life to mitigate long term metabolic and endocrine effects of pre- and post-natal nutritional compromise.
